# Validity and reliability of the Dutch version of the S3-NIV questionnaire to evaluate long-term noninvasive ventilation

**DOI:** 10.1177/14799731241236741

**Published:** 2024-02-29

**Authors:** Charlotte GW Seijger, Bettine AH Vosse, Leandre la Fontaine, Tim Raveling, Nicolle AM Cobben, Peter J Wijkstra

**Affiliations:** 1 Department of Pulmonary Diseases and Home Mechanical Ventilation, University of Groningen, 10173 University Medical Center Groningen, Groningen, The Netherlands; 2 Groningen Research Institute of Asthma and COPD (GRIAC), University of Groningen, 10173 University Medical Center Groningen, Groningen, The Netherlands; 3 Department of Pulmonary Diseases and Home Mechanical Ventilation, 199236 Maastricht University Medical Center+, Maastricht, The Netherlands; 4 Department of Neurology and School for Mental Health and Neuroscience, 199236 Maastricht University Medical Center+, Maastricht, The Netherlands

**Keywords:** Noninvasive ventilation, chronic respiratory failure, patient-reported outcome measures, tele-monitoring

## Abstract

**Objectives:**

Noninvasive ventilation (NIV) is an effective treatment for chronic respiratory failure (CRF). Patient-centered outcomes need to be evaluated regularly and the S^3^-NIV questionnaire seems an applicable tool. We translated this short, self-administered questionnaire into a Dutch version and tested its construct validity and reliability.

**Methods:**

An observational study was conducted, including 127 stable long-term NIV users with CRF or complex sleep related breathing disorders due to different underlying diseases: chronic obstructive pulmonary disease (25%), slowly progressive neuromuscular disorders (35%), rapidly progressive neuromuscular disorders (12%) and ‘other disorders’ (28%) including complex sleep apnea and obesity hypoventilation syndrome. Construct validity and reliability were tested.

**Results:**

The Dutch version of the questionnaire was obtained after a translation and back-translation process. Internal consistency of the total score was good (Cronbach’s α coefficient of 0.78) as well as for the ‘respiratory symptoms’ subdomain and the ‘sleep and side effects’ subdomain (Cronbach’s α coefficient of 0.78 and 0.69, respectively). The reproducibility was excellent with an intraclass correlation of 0.89 (95% CI 0.87-0.93). Construct validity was good for the ‘respiratory symptoms’ subdomain.

**Conclusion:**

The Dutch S^3^-NIV questionnaire is a reliable and valid tool to evaluate symptoms, sleep, and NIV related side effects in long-term NIV users.

## Introduction

Long-term noninvasive ventilation (NIV) is used for patients with chronic hypercapnic respiratory failure (CRF) due to neuromuscular disorders, thoracic cage disorders and pulmonary diseases such as chronic obstructive pulmonary disease (COPD).^1,2^ In addition, NIV can be beneficial for patients with complex sleep-related breathing disorders (SRBD) such as complex sleep apnea and obesity hypoventilation syndrome (OHS).^3,4^ NIV has shown to improve symptoms, quality of life, and survival.^5–7^ In the Netherlands, the number of patients has quadrupled from 5,6/100.000 in 2001 to 22/100.000 almost 20 years later.^8^ Currently, NIV is evaluated mainly by measuring physiological factors, such as blood gasses or transcutaneous gas-exchange measurements, but these factors show weak correlations with patient-centered outcomes such as health-related quality of life (HRQL).^9,10^ Nowadays, several Dutch questionnaires are available to evaluate symptoms and HRQL in patients with CRF. In clinical practice, these are of limited use due to their length and complex scoring algorithms and they do not consider NIV related side effects. The French S^3^-NIV questionnaire provides a patient-completed tool to evaluate patients on long-term NIV, which overcomes the described shortcomings.^11^ It was developed based on a reflective model and contains 11 short questions, which cover three patient-oriented dimensions related to NIV: ‘respiratory symptoms’, ‘sleep quality’, and ‘NIV related side effects’.^11^ Internal consistency, construct validity and reproducibility were shown to be promising.^11^ The S^3^-NIV questionnaire was translated to Portuguese and showed a good internal consistency, but construct validity and test-retest reliability were not assessed.^12^ In both studies, patients with neuromuscular disorders were in minority.^11,12^ However, in the Netherlands, these patients represent the majority of long-term NIV users.^8^ Therefore, this study aims to test the construct validity and reliability of the Dutch version of the S^3^-NIV questionnaire in a large cohort of long-term NIV users including neuromuscular disorders, COPD and other disorders such as OHS, thoracic cage disorders and SRBD. We hypothesize that the construct validity and reliability of the Dutch S^3^-NIV questionnaire are good in all categories of long-term NIV-users.

## Material and methods

### Study design

This prospective observational study was conducted by the home mechanical ventilation centers of the University Medical Center Groningen and the Maastricht University Medical Center in the Netherlands from August 2021 until November 2022. The local Medical Ethics Committee of Groningen concluded that the study protocol falls outside the scope of the Medical Research Involving Human Subjects Act (WMO). The study was conducted according to the applicable research principles and the COSMIN reporting guideline for studies on measurement properties of patient reported outcome measures (PROMs).^13^ Written informed consent was obtained from all included patients. NIV users who visited one of the out-patient clinics for regular evaluation were asked to participate in this study.^14–16^ Adult patients (age ≥18 years) with CRF or complex SRBD, using long-term NIV for at least 3 months were eligible for this study. Based on other respiratory PROMs studies, the aimed number of patients was set on 120.^14–16^ Exclusion criteria were a pulmonary infection, exacerbation COPD or hospitalization in the past 3 months or the inability to understand Dutch. We categorized patients in four groups representing the main indications for long-term NIV in the Netherlands: (1) COPD, (2) slowly progressive neuromuscular disorders (S-NMD), (3) rapidly progressive neuromuscular disorders (R-NMD) and (4) ‘other disorders’ including thoracic cage disorders, OHS and complex SRBD. Baseline patient characteristics, ventilator settings, NIV treatment adherence data, and results of transcutaneous measured carbon dioxide (CO_2_) and oxygen saturation (SenTec DM, SenTec AG, Therwil, Switzerland) were collected from the medical file.^17^ NIV treatment adherence data were collected from the ventilator’s software in all patients. Treatment adherence data were expressed as the number of hours the ventilator was used per day.

### Questionnaires

The original S^3^-NIV questionnaire was developed based on a reflective model, by first selecting all items pertaining to ‘respiratory complaints’ and ‘attendant symptoms and sleep’ from the Severe Respiratory Insufficiency (SRI) questionnaire.^18^ A new dimension regarding comfort and NIV related side effects was obtained via in depth qualitative interviews in 15 patients. The S^3^-NIV questionnaire is a self-administered questionnaire containing 11 items that patients score on a five-point Likert-scale (0: always true; 1: mostly true; 2: sometimes true; 3: mostly untrue; 4: completely untrue) according to how true each statement was perceived in the four preceding weeks. The total score is computed as the average of all answered items multiplied by 2.5. The total scores ranges from 0 to 10, with a higher score indicating the lowest impact of disease and treatment. The ‘respiratory symptoms’ subscore is calculated as the average of answered items 1, 4, 5, six and seven multiplied by 2.5 and the ‘sleep and side effects’ subscore is calculated as the average of answered items 2, 3, 8, 9, 10 and 11 multiplied by 2.5.^11^ Validated Dutch versions of the Clinical COPD Questionnaire (CCQ), the Chronic Respiratory Questionnaire (CRQ), the Maugeri Foundation Respiratory Failure (MRF-28) questionnaire, and the Hospital Anxiety and Depression Scale (HADS) were used to test for construct validity of the S^3^-NIV questionnaire.^14,15,18–21^ Patients filled in the questionnaires at home, on paper or digitally (Castor eClinical Data Management Platform). One week after completing all questionnaires, the S^3^-NIV questionnaire was completed a second time to assess test-retest reliability. The test conditions were similar. A 1-week interval was chosen to minimalize effects on the questionnaires by infections, exacerbations COPD or other medical issues which could interfere with the results. Patients who failed to fill in all items on the questionnaires were excluded from the analyses.

### Dutch translation

The Dutch S^3^-NIV questionnaire was composed using the existing Dutch translation of the seven questions about ‘respiratory symptoms’ and ‘sleep quality’ from the SRI questionnaire.^18,22^ The remaining four questions about NIV related side effects and mask fitting were translated from the presented English variant of the S^3^-NIV questionnaire, respecting international WHO guidelines.^23,24^ First a forward translation was performed and discussed by all investigators followed by a backward translation by an independent English and Dutch native speaking scientific translator. After pre-testing in five patients the final Dutch version was prepared.

### Statistical analysis

All statistical analyses were performed using IBM SPSS software version 28. Normality was determined using the Kolmogorov-Smirnov test. Normally distributed data were described as mean with standard deviation (SD) and comparisons between different patient categories were analyzed using one-way analysis of variance including a post hoc comparison with Bonferroni correction. Non-normally distributed data were described as median and interquartile range [IQR] and comparisons between different patient categories were analyzed using the Kruskal Wallis test including a post hoc comparison with Bonferroni correction. Categorical variables were described as frequencies and compared using Pearson’s chi-squared test. Cronbach’s reliability coefficient α was used to assess the internal consistency of each subscale and Cronbach’s α > 0.7 was considered good.^25^ The test-retest reliability was assessed by the intraclass correlation coefficient (ICC). Spearman correlation coefficient was used to test construct validity between comparable domains of the S^3^-NIV questionnaire to the other questionnaires as well as patient characteristics and ventilator settings. A *p*-value <.05 was considered as statistically significant.

## Results

### Baseline characteristics

Regarding the translation process, no major issues were found and the questionnaire was adapted to the Dutch language (available in the supplementary data). A total number of 127 patients completed all questionnaires. The baseline characteristics are presented in Table 1.

**Table 1.** Baseline demographics and ventilation characteristics.

	COPD *n* = 32	S-NMD *n* = 45	R-NMD *n* = 15	Other disorders *n* = 35	Total *n* = 127
Age, years	68.3 ± 7.2	56.8 ± 14.4	67.5 ± 8.2	65.2 ± 13.6	63.3 ± 12.9*
BMI, kg/m^2^	27.4 ± 4.7	30.2 ± 6.8	24.5 ± 3.4	37.7 ± 11.7	30.8 ± 8.9*
Male sex, n	9 (28.1)	23 (51)	11 (73)	18 (51)	61 (48)*
NIV duration, months	42 [26, 57]	67 [34, 116]	28 [19, 48]	57 [22, 107]	45 [25, 92]*
Treatment adherence, hours/night	07:56 [06:38, 09:07]	08:25 [06:38, 09:07]	09:38 [08:01, 14:00]	08:12 [06:45, 08:53]	08:25* [06:45, 09:12]
IPAP, cmH_2_O	24 [22, 26]	19 [16, 22]	18 [16, 20]	22 [19, 26]	20 [18, 24]*
EPAP, cmH_2_O	6 [5, 7]	7 [6, 9]	7 [6, 8]	9 [7, 11]	7 [6,9]*
P_tcCO2_, kPa	5.6 [5.2, 6.1]	5.4 [4.6, 5.6]	5.5 [5.2, 6.0]	5.4 [4.8, 5.8]	5.5 [5.0, 5.8]*
SO_2_, %	94 [90, 96]	95 [94, 96]	94 [94, 97]	95 [93, 96]	95 [93, 96]*

Abbreviations: NIV = noninvasive ventilation; COPD = chronic obstructive pulmonary disease; S-NMD = slowly progressive neuromuscular disorders; R-NMD = rapidly progressive neuromuscular disorders; BMI = body mass index; IPAP = inspiratory positive airway pressure; EPAP = expiratory positive airway pressure; P_tcCO2_ = nocturnal transcutaneous measured carbon dioxide level; SO_2_ = nocturnal oxygen saturation. Note: data expressed as mean ± SD, frequency (%), median [IQR]. *Statistically significant differences between disease groups (*p* < .05).

The median time since NIV initiation was 45 months (IQR 25, 92) and patients were adherent to long-term NIV, with nocturnal transcutaneous CO_2_ monitoring showing normocapnia in all groups indicating effective treatment with NIV. The S-NMD group included patients with myotonic dystrophy type 1 (15), hemi- (5) or complete diaphragmatic paralysis (4), neuralgic amyotrophy (3), spinal muscular atrophy (2), facioscapulohumeral dystrophy (2), myasthenia gravis (2), Duchenne muscular dystrophy (2), limb-girdle muscular dystrophy (2), Pompe disease (2), and other neuromuscular disorders (6). The R-NMD group included patients with ALS (12), progressive spinal muscular atrophy (2) and primary lateral sclerosis (1). The ‘other disorders’ group consisted of patients with OHS (20), thoracic cage disorders (8) and SRBD (7).

### Distribution of the S^3^-NIV questionnaire

**Figure 1.**
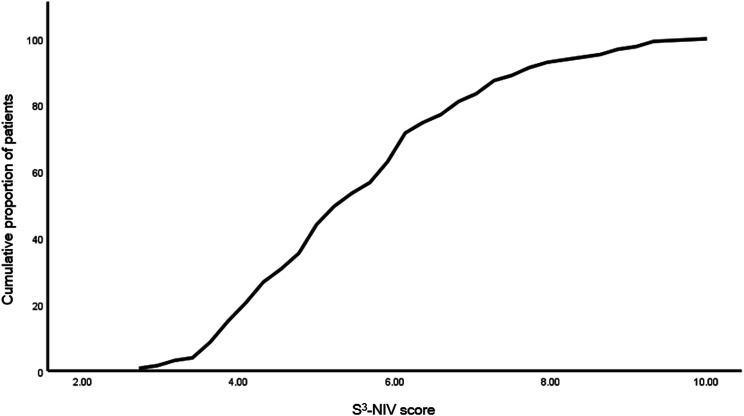
Cumulative distribution of the S3-NIV questionnaire total score for all participants.

**Figure 2.**
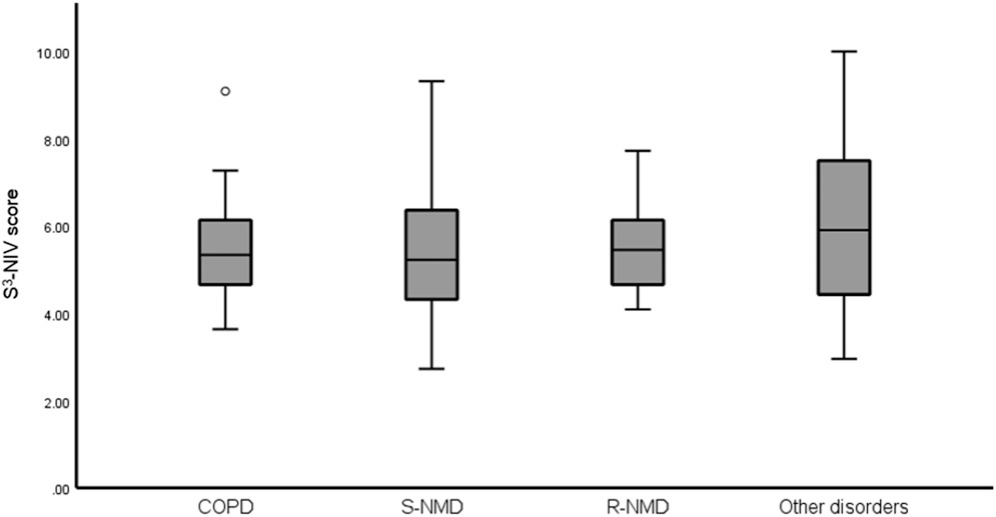
Distribution of the S3-NIV total score stratified per diagnosis. Note the outlier score in the COPD group depicted with an open circle. Abbreviations: COPD=chronic obstructive pulmonary disease; S-NMD=slowly progressive neuromuscular disorders; R-NMD=rapidly progressive neuromuscular disorders.

In Figures 1 and 2, the distributions of the S^3^-NIV scores are expressed. The entire scaling range was used without floor or ceiling effect in any subgroup. Of the total 127 patients, 80% used 38.7% of the total range, 10% had a score of <3.86 and 10% had a score >7.73. The median total S^3^-NIV score was 5.5 (IQR 4.3, 6.6). In Table 2, the S^3^-NIV scores per subgroup are shown.

**Table 2.** S^3^-NIV (sub) scores per category.

	Total score	Respiratory symptoms subscore	Sleep and side effects subscore
COPD	5.3 [4.6, 6.1]	4.8 [3.5, 5.9]	6.3 [5.4, 7.1]
S-NMD	5.2 [4.2, 6.5]	4.5 [3.5, 6.0]	5.8 [4.8, 7.1]
R-NMD	5.5 [4.5, 6.1]	4.5 [4.0, 6.0]	6.3 [5.4, 7.5]
Other disorders	5.9 [4.3, 7.7]	5.5 [3.5, 8.0]	6.3 [5.0, 7.1]
Total	5.5 [4.3, 6.6]	4.5 [3.5, 6.0]	6.3 [5.0, 7.1]

Abbreviations: COPD = chronic obstructive pulmonary disease; S-NMD = slowly progressive neuromuscular disorders; R-NMD = rapidly progressive neuromuscular disorders. Note: data expressed as median [IQR].

The S-NMD group and the ‘other disorders’ group had larger standard deviations than the COPD and R-NMD group, but there were no statistically significant differences between the groups.

### Reliability

The internal consistency of the total score and subdomains was good, Cronbach α of 0.78 for the total score and the ‘respiratory symptoms’ subdomain, and 0.69 for the ‘sleep and side effects’ subdomain. The S^3^-NIV questionnaire was completed again 1 week after the initial assessment by 123 patients out of 127. Despite reminders, four patients did not complete the S^3^-NIV questionnaire for a second time. The ICC was 0.89 (95% CI 0.87-0.93), which indicates excellent test-retest reliability.

### Construct validity of the S^3^-NIV questionnaire

Table 3 shows the outcomes of the Spearman’s rank correlation test between the S^3^-NIV questionnaire and the other questionnaires (CRQ, MRF-28, HADS, and CCQ).

**Table 3.** Correlations between subdomains S^3^-NIV questionnaire and CRQ, MRF-28, HADS and CCQ.

	S^3^−NIV questionnaire subdomains		Total
	Respiratory symptoms	Sleep and side effects	
CRQ subdomains			
Dyspnea	0.50***	−0.05	0.33**
Fatigue	0.60***	0.29***	0.53***
Emotional	0.59***	0.28**	0.52***
Mastery	0.48***	0.19*	0.40***
MRF-28 subdomains			
Daily activity	−0.30***	0.10	−0.12
Cognitive function	−0.55***	−0.27**	−0.49***
Invalidity	−0.34***	−0.22**	−0.33***
HADS subdomains			
Anxiety	−0.51***	−0.31***	−0.49***
Depression	−0.53***	−0.23**	−0.44***
CCQ subdomains			
Symptoms	−0.75***	−0.33***	−0.67***
Functional state	−0.55***	−0.27**	−0.48***
Mental state	−0.54***	−0.24**	−0.47***

Abbreviations: CRQ = chronic respiratory questionnaire; MRF-28 = Maugeri respiratory failure questionnaire; HADS = hospital anxiety and depression scale; CCQ = clinical COPD questionnaire. Note: * *p*-value <.05; ** *p*-value <.01; *** *p*-value <.001.

High correlations were found between the ‘respiratory symptoms’ subdomain with several subdomains of the tested questionnaires, such as the CCQ ‘symptoms’ and the CRQ ‘dyspnea’ subdomain (rho = −0.75, *p* < .001, rho = 0,50, *p* < .001 respectively). On the contrary, only weak to moderate correlations were found between the ‘sleep and side effects’ subdomain with the other questionnaires. Baseline characteristics such as age, body mass index, ventilator settings, treatment adherence to NIV and CO_2_ values were not correlated to the S^3^-NIV scores (data not shown).

## Discussion

This study shows that the Dutch version of the S^3^-NIV questionnaire has a good construct validity and reliability in a large cohort of patients using long-term NIV due to different etiologies. The S^3^-NIV questionnaire can be used in the entire long-term NIV population in the Netherlands and other Dutch speaking countries.

The reliability was investigated by assessing the internal consistency and test-retest reliability. The Cronbach’s α coefficient of 0.78 in the present study is comparable to the original study and the first available translated (Portuguese) version (respectively 0.84 and 0.76).^11,12^ In addition, this is the first study that confirms an excellent reproducibility with an ICC of 0.89 (95% CI 0.87-0.93), which is higher than in the original study (0.72, 95% CI 0.54-0.84).^11^ Validity was tested by comparison of the S^3^-NIV questionnaire with the CRQ, MRF-28, HADS and CCQ questionnaires. The construct validity for the ‘respiratory symptoms’ subdomain was found to be good. However, it was difficult to test the construct validity for the ‘sleep and side effects’ subdomain due to the absence of a comparable (Dutch) questionnaire. For the development of the S^3^-NIV questionnaire the Quebec Sleep Questionnaire (QSQ) was used to validate the ‘sleep and side effects’ subdomain. Unfortunately, the QSQ is not available in Dutch and there is currently no other (Dutch) questionnaire covering this domain. Moreover, there is no questionnaire containing questions on NIV related side effects. Evaluation of side effects of long-term NIV is essential because side effects negatively influence treatment adherence and are the main reason that patients discontinue NIV.^26^ Taken together, the S^3^-NIV questionnaire meets the need of evaluating patients-centered outcomes in long-term NIV users in a short and easy way. Additionally, long-term NIV is more frequently initiated at the patients’ home using tele-monitoring and the S^3^-NIV questionnaire may be a promising tool that can facilitate tele-monitoring.^27–30^

The present study included more patients with neuromuscular diseases compared to the previous studies with the S^3^-NIV questionnaire, therefore better reflecting the Dutch long-term NIV population.^5,8^ Remarkably, we found lower S^3^-NIV scores in all patient groups compared to previous studies, indicating a higher impact of disease and treatment.^11,12^ Unfortunately, additional information, such as pulmonary function tests and functional status were not available to explain these differences. A possible reason could be that this Dutch population reflects more severely affected patients, because within Europe, there are wide variations in selection criteria for initiating NIV.^5^ In the Netherlands, a national guideline describes strict criteria about referral of patients and initiation of NIV, which might explain that the selected group is more severely affected.^31^

There might be some limitations to this study. Firstly, our study population represent the Dutch long-term NIV population well, but it might differ from populations elsewhere in the world, although it seems unlikely that this cohort is fundamentally different from other cohorts.^4,5^ Secondly, we have chosen to translate the presented English version of the S^3^-NIV questionnaire instead of the original French version as seven out of 11 questions were already available in the Dutch SRI questionnaire. To incorporate the S^3^-NIV questionnaire in daily care a future longitudinal study is needed to assess cut off values and to determine the minimal clinically important difference of the questionnaire. This is needed to monitor both worsening of the underlying disorder as well as evaluating the effects of modifications in NIV settings or interfaces. In order to meet the growing demand of NIV users, who are more frequent subjected to home initiation in the Netherlands, this short and easy Dutch S^3^-NIV questionnaire is an important additional tool in (tele-)monitoring.^27–30^

## Conclusion

In this study, the Dutch version of the S^3^-NIV questionnaire has been shown to be a simple, valid and reliable tool to evaluate symptoms, sleep and NIV related side effects in long-term NIV users.

## Supplemental Material

Supplemental Material - Validity and reliability of the Dutch version of the S3-NIV questionnaire to evaluate long-term noninvasive ventilation Supplemental Material for Validity and reliability of the Dutch version of the S3-NIV questionnaire to evaluate long-term noninvasive ventilation by Charlotte GW Seijger, Bettine AH Vosse, Leandre la Fontaine, Tim Raveling, Nicolle AM Cobben and Peter J Wijkstra in Chronic Respiratory Disease

## Data Availability

Data can be made available by the corresponding author upon request.
