# Supplementary material for: Validity and reliability of the Dutch version of the S3-NIV questionnaire to evaluate long-term noninvasive ventilation
Source: Chron Respir Dis. 2024 Feb 29;21:14799731241236741. doi: 10.1177/14799731241236741 (PMC10906045; doi:10.1177/14799731241236741)
Supplement: Supplemental Material - Validity and reliability of the Dutch version of the S3-NIV questionnaire to evaluate long-term noninvasive ventilation [file sj-pdf-1-crd-10.1177_14799731241236741.pdf]

Vragenlijst thuisbeademing, de 'S3-NIV' vragenlijst

Patiënt / Deelnemernummer ……………………………………………………..

Invuldatum: ……………………………………………………..

Deze vragenlijst gaat over uw long-klachten, slaap kwaliteit en bijwerkingen van thuisbeademing.

Hoe ervaarde u de ***afgelopen 4 weken***?
Graag voor IEDERE vraag een kruisje plaatsen bij het antwoord dat het meest passend voor u is.

	Zeer mee eens (0)	Wel mee eens (1)	Beetje mee eens (2)	Niet mee eens (3)	Helemaal niet mee eens (4)
1. Tijdens het eten heb ik adem tekort.	☐	☐	☐	☐	☐
2. Ik heb dikwijls hoofdpijn.	☐	☐	☐	☐	☐
3. Ik word 's nachts wakker door adem tekort.	☐	☐	☐	☐	☐
4. Ik ben vaak kortademig.	☐	☐	☐	☐	☐
5. Tijdens het praten heb ik adem tekort.	☐	☐	☐	☐	☐
6. Mijn luchtwegen zijn dikwijls met slijm gevuld.	☐	☐	☐	☐	☐
7. Bij lichamelijke belasting heb ik adem tekort.	☐	☐	☐	☐	☐
8. Ik heb last van (lucht) lekkage langs het masker.	☐	☐	☐	☐	☐
9. Mijn masker zit niet prettig.	☐	☐	☐	☐	☐
10. Ik krijg te veel lucht van de (beademings) machine.	☐	☐	☐	☐	☐
11. Door het gebruik van de beademing ervaar ik een droge neus en/of mond.	☐	☐	☐	☐	☐

Elise Dupuis-Lozeron et al. Eur Respir J
2018;52:1801182

Appendix B: Subject Consent Form

A short 'S^3-NIV questionnaire' to measure effects of chronic non-invasive ventilation

- I have read the subject information form. I was also able to ask questions. My questions have been answered to my satisfaction. I had enough time to decide whether to participate.

- I know that participation is voluntary. I know that I may decide at any time not to participate after all or to withdraw from the study. I do not need to give a reason for this.

- I give permission for my treating specialist(s) to be informed that I am participating in this study.

- I give permission for information to be requested from my treating specialist about my medical issues.

- I give permission for the collection and use of my data to answer the research question in this study.

- I know that some people may have access to all my data to verify the study. These people are listed in this information sheet. I consent to the inspection by them.

- I □ do □ do not consent to keeping my personal data longer and to use it for future research in the field of chronic non-invasive ventilation.

- I □ do

□ do not consent to being contacted again after this study for a follow-up study.

- I want to participate in this study.

Name of study subject:

Signature: Date: __ / __ / __

I hereby declare that I have fully informed this study subject about this study.

If information comes to light during the course of the study that could affect the study subject's consent, I will inform him/her of this in a timely fashion.

Name of investigator (or his/her representative):

Signature: Date:__ / __ / __

The study subject will receive the full information sheet, together with a signed copy of the consent form.
